# The Effects of Exercise on Synaptic Stripping Require Androgen Receptor Signaling

**DOI:** 10.1371/journal.pone.0098633

**Published:** 2014-06-02

**Authors:** Caiyue Liu, Patricia J. Ward, Arthur W. English

**Affiliations:** 1 Department of Plastic and Reconstructive Surgery, Changzheng Hospital, Second Military Medical University, Shanghai, China; 2 Department of Cell Biology, Emory University School of Medicine, Atlanta, Georgia, United States of America; The University of Western Australia, Australia

## Abstract

Following peripheral nerve injury, synapses are withdrawn from axotomized motoneurons. Moderate daily treadmill exercise, which promotes axon regeneration of cut peripheral nerves, also influences this synaptic stripping. Different exercise protocols are required to promote axon regeneration in male and female animals, but the sex requirements for an effect of exercise on synaptic stripping are unknown. In male and female C57BL/6 mice, the sciatic nerve was transected in the mid-thigh. Mice were then exercised five days per week for two weeks, beginning on the third post-transection day. Half of the exercised mice were trained by walking slowly (10 M/min) on a level treadmill for one hour per day (continuous training). Other mice were interval trained; four short (two min) sprints at 20 M/min separated by five minute rest periods. A third group was untrained. The extent of synaptic contacts made by structures immunoreactive to vesicular glutamate transporter 1 and glutamic acid decarboxylase 67 onto axotomized motoneurons was studied in confocal images of retrogradely labeled cells. Both types of presumed synaptic contacts were reduced markedly in unexercised mice following nerve transection, relative to intact mice. No significant reduction was found in continuous trained males or interval trained females. Reductions in these contacts in interval trained males and continuous trained females were identical to that observed in untrained mice. Treatments with the anti-androgen, flutamide, blocked the effect of sex-appropriate exercise on synaptic contacts in both males and females. Moderate daily exercise has a potent effect on synaptic inputs to axotomized motoneurons. Successful effects of exercise have different requirements in males and females, but require androgen receptor signaling in both sexes.

## Introduction

Axons in injured peripheral nerves have the capacity for regeneration, but functional recovery after peripheral nerve injury is poor [1], [2]. Following transection of a peripheral nerve, a withdrawal of nearly half of the synaptic inputs onto the somata and proximal dendrites of the axotomized motoneurons is found (reviewed in [3]). Both excitatory and inhibitory inputs are withdrawn. Over time, many of these inputs are restored, but those containing vesicular glutamate transporter 1 (VGLUT1), and arising mainly from primary afferent neurons [4], [5] remain withdrawn [3]. The irreversible withdrawal of synapses arising from these stretch-sensitive afferents is thought to be an important factor contributing to the loss of the stretch reflex in self-reinnervated muscles [6] and may contribute significantly to the poor functional recovery found clinically after recovery from peripheral nerve injury.

We have shown that moderate exercise in the form of daily treadmill training after peripheral nerve injury produced a substantial enhancement of axon regeneration [7]. We also found a profound sex difference in the requirements for exercise to promote axon regeneration in cut peripheral nerves [8]. Continuous training (an hour of daily slow walking) enhances axon regeneration in male mice, but not in female mice while interval training (a series of interrupted short sprints) is effective in female and not male mice. Following sciatic nerve transection, we also showed that the expected loss of contacts onto motoneurons by synaptic structures was not observed in interval trained female mice [9]. One goal of this study was to examine whether a similar sex difference in the requirements for the effects of exercise on synaptic withdrawal was found.

Androgens are well known to play important roles in recovery following peripheral nerve injury. Treatments with testosterone induced acceleration of functional recovery from lower limb paralysis following sciatic nerve crush in the rat [10], [11]. Such treatments are thought to promote elongation of regenerating axons, as they decrease time to recovery after both facial nerve crush injury [12], [13] and recurrent laryngeal nerve injury [14]. In the central nervous system (CNS), testosterone propionate treatment significantly attenuated the amount of synaptic withdrawal and the decrease in average length of the remaining synapses in an adult male hamster model of facial nerve transection [15]. A similar reduction of synapse loss was found after testosterone treatment in male rats exposed to chronic stress [16]. All of these effects of androgens require signaling through the androgen receptor. Exposure to flutamide, a potent non-steroidal anti-androgen, completely abolished the testosterone-induced enhancing effects on facial nerve regeneration [17].

Castration eliminates the effect of continuous training on enhancement of axon regeneration in male mice. Treatments of unexercised female mice with an inhibitor of aromatase, an enzyme that converts androgens or their precursors into estradiol, enhanced axon regeneration significantly [8]. Treatments of both male and female mice with flutamide blocked the enhancing effect of exercise on axon regeneration in peripheral nerves completely [18]. Based on these results, we hypothesized that androgens also would be required for the effects of exercise in restoring withdrawn synapses from axotomized motoneurons in both males and females.

To test this hypothesis, we used flutamide to block signaling through the androgen receptor and then evaluated the extent of contacts made by excitatory (VGLUT1) and inhibitory (glutamic acid decarboxylase, GAD67) immunoreactive terminals on the somata and proximal-most dendrites of both intact and axotomized motoneurons in exercised and unexercised female and male mice. We report here that exercise has similar effects on synaptic contacts to axotomized motoneurons in male and female mice, that the requirements for exercise to produce these effects are different in male and female mice, and that this effect of exercise is blocked completely by flutamide treatments in both males and females.

## Materials and Methods

### Ethical Statements

All experimental procedures conformed to the Guidelines for the Use of Animals in Research of the Society for Neuroscience and were approved by the Institutional Animal Care and Use Committee of Emory University (Protocol #2002017).

### Animals

Experiments were conducted on 64 wild type (C57BL/6J) adult (>2 months old) mice weighing 18 g–32 g. Mice were randomly divided into six groups for both females and males as follows: (1): sciatic nerve intact; (2): right sciatic nerve transection without treadmill exercise; (3): right sciatic nerve transection plus treadmill exercise; (4): sciatic nerve intact plus flutamide treated; (5): right sciatic nerve transection plus flutamide treated; (6): right sciatic nerve transection plus treadmill exercise and flutamide treated. For the exercised and untreated animals (group 3), two different exercise protocols, continuous and interval (see below) were applied to males and females. The numbers of mice studied were based on a power sample size estimate [19] using a power of 0.8, alpha = 0.05 and the inter-animal standard deviations (0.5–0.8%) and changes in synaptic coverage reported in the literature [3], [20] as a reasonable expectation of anticipated differences between groups. For N = 4, as in most of the groups studied, a true difference in means of 1.66% was estimated. The numbers of mice in each group are shown in Table 1.

**Table 1:** Summary of Animals Studied by Treatment Condition.

	Exercise				
Sciatic n. Transection	Continuous	Interval	Flutamide	Sex	N
No	No	No	No	M	8
No	No	No	No	F	8
No	No	No	Yes	M	4
No	No	No	Yes	F	4
Yes	No	No	No	M	4
Yes	No	No	No	F	4
Yes	Yes	No	No	M	4
Yes	No	Yes	No	M	4
Yes	Yes	No	No	F	4
Yes	No	Yes	No	F	4
Yes	No	No	Yes	M	4
Yes	No	No	Yes	F	4
Yes	Yes	No	Yes	M	4
Yes	No	Yes	Yes	F	4
TOTAL					64

### Surgical procedures

All mice were subjected to two survival surgeries while anesthetized with isoflurane. Three days prior to sciatic nerve transection surgery, the motoneuron cell bodies were labeled bilaterally by injecting the retrograde tracer, cholera toxin beta subunit (CTB) conjugated with Alexafluor 555 (Invitrogen) (1 µg/µl in distilled water), into the right and left gastrocnemius and tibialis anterior muscles respectively (1 µl each) of isoflurane anesthetized mice [21]. After the tracer had been taken up and transported to motoneuron cell bodies, the animals were again anesthetized and the right sciatic nerve was cut in the mid-thigh. The left sciatic nerve remained intact. To rule out any effect of trophic molecules in the distal segment of the cut nerve [22], [23] or from the muscle targets of motoneurons, we chose not to repair the cut nerves.

To assess the requirement for androgen receptor signaling on the effect of exercise on synaptic withdrawal after injury, flutamide, an androgen receptor antagonist, was applied systemically in a sustained release dosage form [24]. Capsules composed of 15 mm long Silastic tubing (1.57 mm i.d.; 3.18 mm o.d.; Dow Corning Corp., Midland, MI) were packed with 5 mm of flutamide powder (2-methyl-*N*-[4-nitro-3-(trifluoromethyl)phenyl]-propanamide; Sigma Aldrich, Seelze, Germany) and flanked on each side by 5 mm wooden stint segments. The ends of the capsules were then covered externally by Medical Adhesive A (Dow Corning). Capsules were soaked in normal saline solution at 37°C for 24 hours prior to implantation to prime the flutamide powder for release. Using this approach, the flutamide was released gradually, by diffusing through the tubing, the rate of release being roughly proportional to the capsule surface area [25]. Two flutamide capsules (ca. 16.8 mg of flutamide) were implanted subcutaneously in each animal three days prior to the onset of treadmill training. We have shown previously that this approach is effective in blocking androgen receptors [18].

### Treadmill training

Beginning on the third day post-nerve transection, mice were treadmill trained for five days a week for two weeks, using either an interval or continuous paradigm [7]. We have previously reported a sex difference in the effect of treadmill training in promoting axon regeneration from cut peripheral nerves [8]. To evaluate whether the same difference in the effect of treadmill training on synaptic withdrawal was found, different groups of male and female mice were trained either continuously for one hour at a slow treadmill speed of 10 m/min (continuous training) or as four repetitions at a faster speed of 20 m/min for two minutes with a five-minute rest periods interspersed (interval training).

### Tissue harvesting and preparation

Two weeks after nerve transection, mice were euthanized with an overdose of pentobarbital (150 mg/kg) and perfused through the heart with saline, followed by periodate-lysate-paraformaldehyde fixative [26]. The L3-L5 segments of spinal cord were removed and stored overnight in 20% sucrose solution at 4°C for cryoprotection. Serial transverse sections were cut at 20 µm on a cryostat and mounted directly onto subbed slides. Standard immunohistological procedures were performed to detect the synaptic vesicle proteins glutamic acid decarboxylase protein 67 (GAD67) (Millipore #MAB5406, USA) or vesicular glutamate transporter 1 (VGLUT1) (Synaptic Systems GmbH #TO30326, Germany). Glutamic acid decarboxylase (GAD), the rate-limiting enzyme in the synthesis of gamma amino butyric acid (GABA), the main inhibitory neurotransmitter in the central nervous system, is found as two isoforms, known by their molecular weights as GAD67 and GAD65. The majority of GABAergic boutons in the ventral horn of the spinal cord are strongly immunoreactive for GAD67 and originate from interneurons in the spinal cord [27]. Synaptic terminals onto motoneurons that are immunoreactive for VGLUT1 have been shown to originate mainly from primary afferent neurons [4], [5]. We assumed that because the proportions of inputs immunoreactive for VGLUT1 arising from other sources is quite small, any contributions that they might make to the findings presented below were not considered. Sections were incubated in primary antibodies, diluted in 1∶2000 for rabbit anti-VGLUT1, 1∶200 for mouse anti-GAD67, for 24 hours at room temp. After washing in 0.1 M PBS for 3 times, the sections were incubated with secondary antibodies (Alexafluor 488, goat anti-rabbit IgG or goat anti-mouse IgG) for 2 hours at room temp to detect immunofluorescence. All the incubations and reactions were separated by 3×10 min washes in 0.1 M PBS.

### Microscopy and imaging analysis

All analyses of tissue sections in this study were performed in such a way that the person collecting images and taking measurements from the collected images was unaware, while performing these tasks, of the experimental conditions applied to the tissues. Histological sections of L3–L5 spinal cord were viewed with a laser scanning confocal microscope (Zeiss LSM510). Labeled motoneurons were selected for study only if they contained labeling that filled the somata and extended into the proximal dendrites and their cell borders could be recognized. Twenty to 40 high magnification (40x) RGB images of individual labeled motoneuron cell bodies were obtained, at a confocal Z-dimension thickness of 1 µm, from each side of each spinal cord studied.

**Figure 1:**
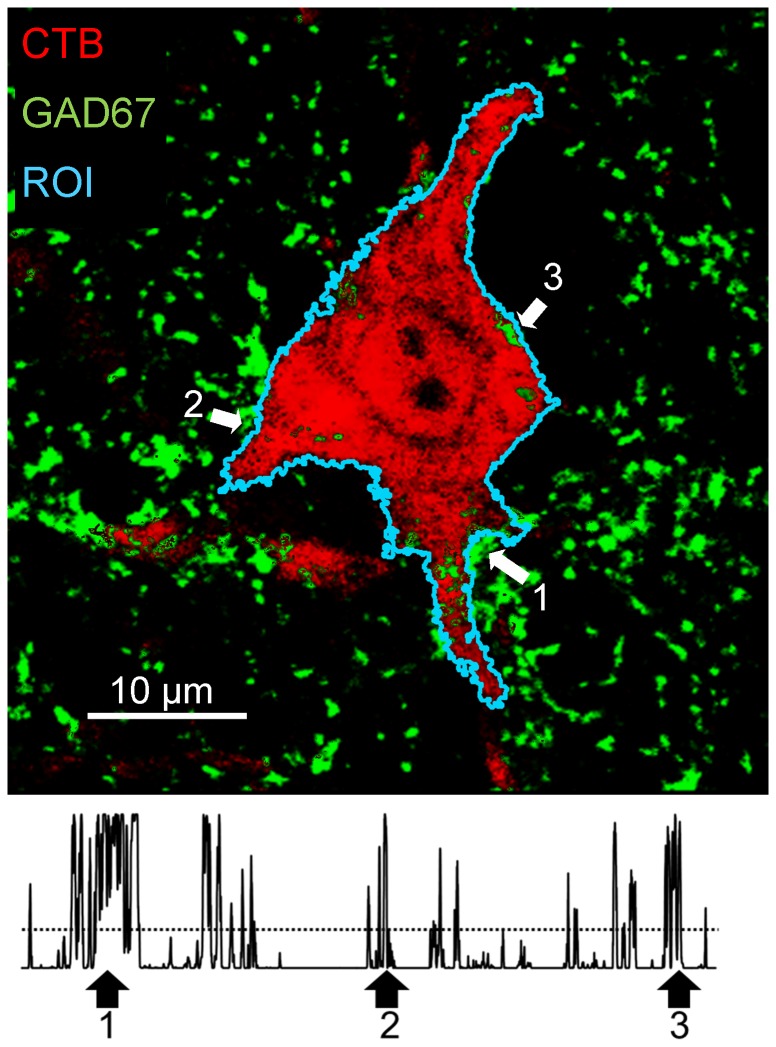
A single retrogradely labeled motoneuron (red) is shown to demonstrate the measurements made throughout this study. This image is from a histological section reacted with an antibody to glutamic acid decarboxylase 67 (GAD67, green). A region of interest (ROI) around the perimeter of this profile of the motoneuron visible in this confocal image was created (cyan line) and the green fluorescence intensity was measured beneath the ROI. In the resulting plot profile (bottom), a threshold intensity (horizontal dashed line) was established, based on the red intensity within the ROI. Intensity values above this threshold were assumed to represent contacts of the motoneuron soma and structures immunoreactive for synapse-specific antigens. Arrows are placed at three examples of the several clusters of contacts in the image and the corresponding position of the plot profile.

All measurements from these images were made using ImageJ software, using a method analogous to that of Wang et al, [28]. A region of interest (ROI) was created around the perimeter of each motoneuron studied (Fig. 1, top), and used to study contacts made by structures immunoreactive to synapse-specific proteins. Because we considered that the identification of this ROI was critical to all subsequent analyses, we tried to make the determination of cell outline as objective as possible. We used the thresholding function of ImageJ while viewing only the red channel of the RGB images; the one containing images of the retrograde label in the motoneuron, because the contrast between the border of the cells studied and the neuropil is so substantial. Cells in which such contrast in the red channel was not entirely clear were not studied. Once a ROI was established, a plot profile (Fig. 1, bottom) was then created to measure the fluorescence intensity, along the region of interest, in the green channel of the RGB image in which immunoreactivity for different synaptic vesicle proteins was displayed. Then, the mean fluorescence intensity (plus the 95% confidence interval) in this latter channel was measured *within* the ROI and used to establish the background fluorescence intensity threshold for that cell. We assumed that any fluorescence within the ROI on this channel was non-specific. We assumed that any fluorescence intensity values in the profile plot for each cell studied that were greater than this threshold represented contact of the motoneuron by structures immunoreactive for the synaptic vesicle protein studied. In Figure 1 (Top) the locations of three (among several) such locations along the ROI are shown. The percentage of the perimeter of the cell profile in which contact by such VGLUT1+ or GAD67+ immunoreactive structures was found was calculated and expressed as percent synaptic coverage. Mean values of percent synaptic coverage by VGLUT1+ and GAD67+ structures were determined for each mouse studied.

We assume that these structures immunoreactive for synapse-specific proteins that are in very close proximity to the outline of the labeled motoneurons represent synaptic contacts made on the motoneurons. This assumption has been supported by others. Immunoreactive structures identified using the same antibodies as used here have been shown to contain synaptic active zones and, after extensive high magnification reconstruction, to be in very close contact to the somata of intracellularly filled motoneurons [29]. However, without higher resolution images available through electron microscopy, we acknowledge that we cannot rule out that at least some of the contacts we have studied might be separated from the motoneuron soma by ultra-fine processes of glial cells.

### Statistical analysis

All data are expressed as means ± SEM. Significance of differences in mean percent synaptic coverage between groups and between cohorts within groups was evaluated using analysis of variance (ANOVA) (Statistica, StatSoft, Tulsa, OK). When the omnibus test for significance was met (p<0.05), paired post-hoc testing (Tukey's honest significant differences (HSD)) was applied.

## Results

We studied the effect of moderate daily exercise on changes in synaptic contacts made by terminals of afferent neurons immunoreactive for the synapse-specific proteins VGLUT1 and GAD67 onto motoneurons following peripheral nerve injury. Structures containing VGLUT1 are assumed to be excitatory terminals arising from primary afferent neurons [4], [5]. Most of them are permanently withdrawn following peripheral nerve transection [3]. Terminals containing GAD67 arise from inhibitory interneurons in the spinal cord. They are withdrawn transiently following peripheral nerve transection [3]. Although these are not all of the different types of synapses that make contact with spinal motoneurons, we studied them as different models of synapses that are withdrawn following nerve injury.

### Sciatic nerve transection results in synaptic withdrawal in both male and female mice

**Figure 2:**
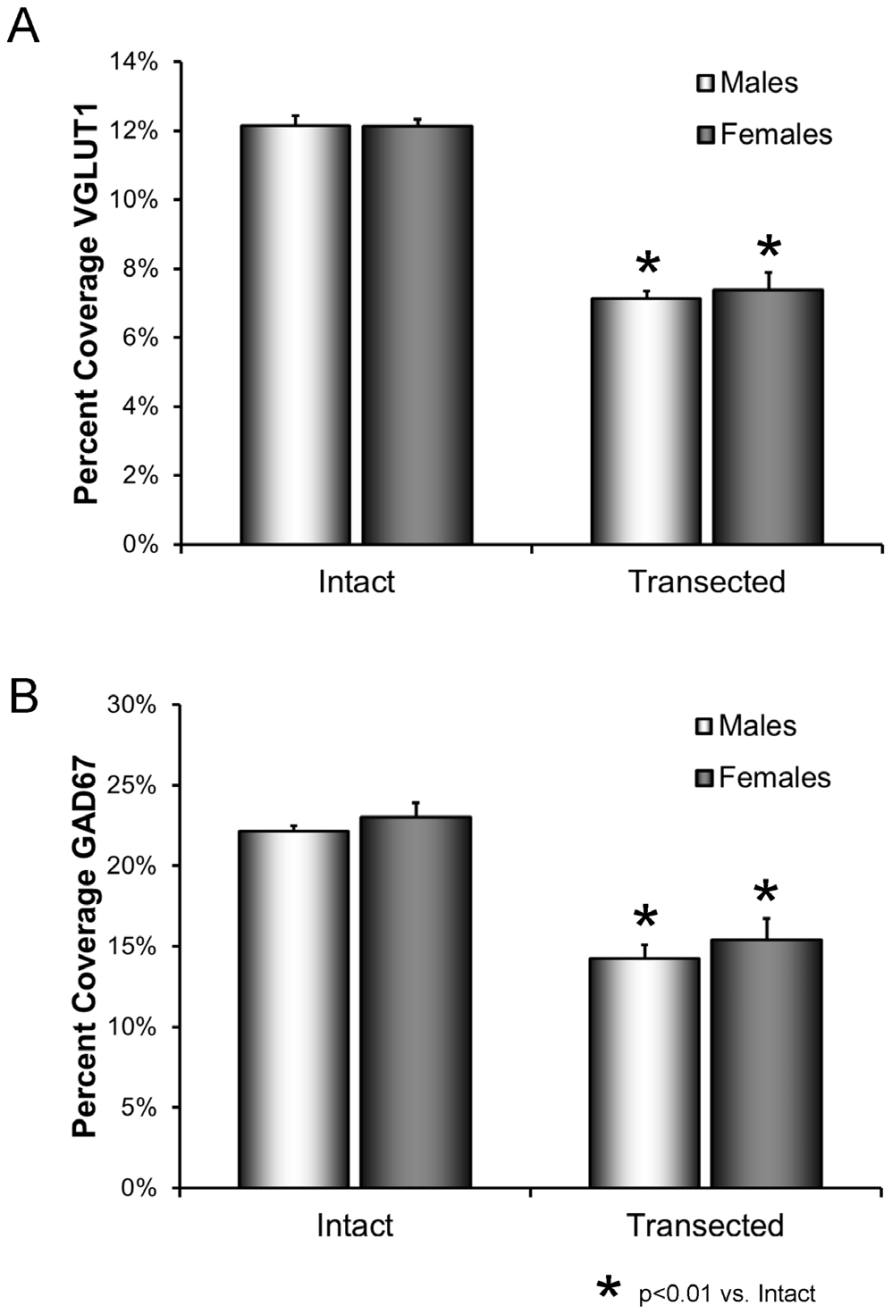
A. Mean (±SEM) percent coverage of motoneuron somata by structures immunoreactive for VGLUT1 in intact male and female mice and male and female mice two weeks following sciatic nerve transection. B. Mean (±SEM) percent coverage of motoneuron somata by structures immunoreactive for GAD67 in intact male and female mice and male and female mice two weeks following sciatic nerve transection.

The extent of contacts made by structures immunoreactive for the synapse-specific proteins VGLUT1 and GAD67 onto the somata and proximal-most dendrites of retrogradely labeled motoneurons were studied in intact male and female mice and in male and female mice two weeks after transection of the sciatic nerve. The resulting measurements are expressed as percent synaptic coverage. Following transection of the sciatic nerve, synapses are withdrawn from the somata of the axotomized motoneurons. The extent of this reduction in percent synaptic coverage is shown quantitatively, as means (± SEM) in Figure 2. The results of ANOVA for the effects of sex and transection were significant, both for VGLUT1 (F_3,20_ = 38.77, p<0.01) and GAD67 (F_3,20_ = 32.92, p<0.01). Statistically significant reductions in percent synaptic coverage by VGLUT1 immunoreactive (IR) structures following sciatic nerve transection were found in both males and females (HSD, p<0.01 for both) (Fig. 2A). No significant sex difference was found. Coverage by GAD67- IR structures was reduced after transection (HSD, p<0.01 in both males and females) (Fig 2B) and no significant sex difference was encountered.

### Sex-appropriate exercise influences the withdrawal of synaptic terminals from motoneurons after peripheral nerve injury

We have shown that enhancing axon regeneration in cut peripheral nerves requires quite different exercise protocols in male and female mice [8]. Regeneration is enhanced in male mice by daily walking for one hour at a slow treadmill speed (10 M/min), termed continuous training. Females respond similarly only to more intense interval training (four repetitions of two minute sprints at 20 M/min, separated by 5 minute rest periods), a pattern of exercise that resembles that used during voluntary wheel running in mice [30]. Thus we wanted to evaluate the effects of exercising male and female mice using these different training protocols.

**Figure 3:**
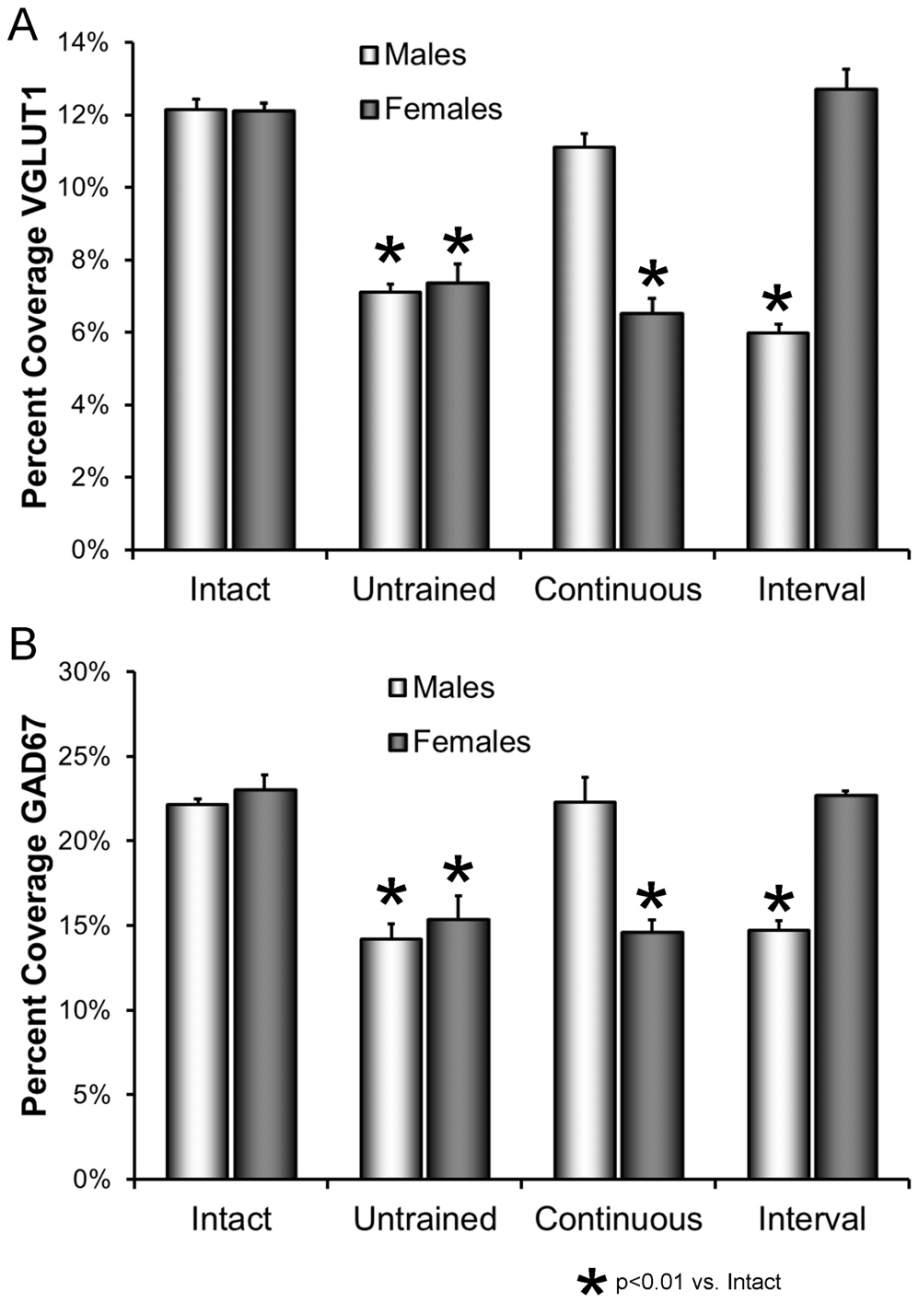
Effects of treadmill exercise on synaptic withdrawal from axotomized motoneurons. A. The mean (±SEM) proportions of motoneuron somata in contact with structures immunoreactive for VGLUT1 are shown for intact mice and in mice two weeks after sciatic nerve transection that were either untrained or exercised using a continuous or interval training protocol. Separate bars are shown for data from males and females. B. As in A, except for contacts immunoreactive to GAD67. In both panels, the data for intact and untrained animals are the same as shown in Figure 2.

Mean (±SEM) percent synaptic coverage by VGLUT1-IR and GAD67-IR structures is shown for the different groups in Figure 3. Data for intact and untrained mice, described above and shown in Figure 2, are included to make comparisons easier. For both VGLUT1-IR and GAD67-IR contacts, the results of the omnibus test from the ANOVA were significant (VGLUT1, F_7,33_ = 49.58, p<0.01; GAD67, F_7,33_ = 24.78, p<0.01). In male mice that were exercised with a continuous training protocol and female mice that were treated with an interval training protocol, no significant reduction in percent synaptic coverage was found for either marker, relative to that for intact mice (HSD, n.s.). However, both in male mice that were interval trained and in female mice that were trained according to a continuous training protocol, a quite different outcome was observed. In both cases a significant reduction in percent synaptic coverage was found for both VGLUT1 and GAD67, relative to intact mice (HSD, p<0.01 for both), and neither was significantly different from untrained mice of the same sex (HSD, n.s.). Exercise has the same sex-dependent requirements for effects on synaptic inputs to spinal motoneurons as found for the effects of exercise in promoting axon regeneration in the periphery.

### Treatments with flutamide block the effects of exercise on synaptic withdrawal after peripheral nerve injury in both male and female mice

**Figure 4:**
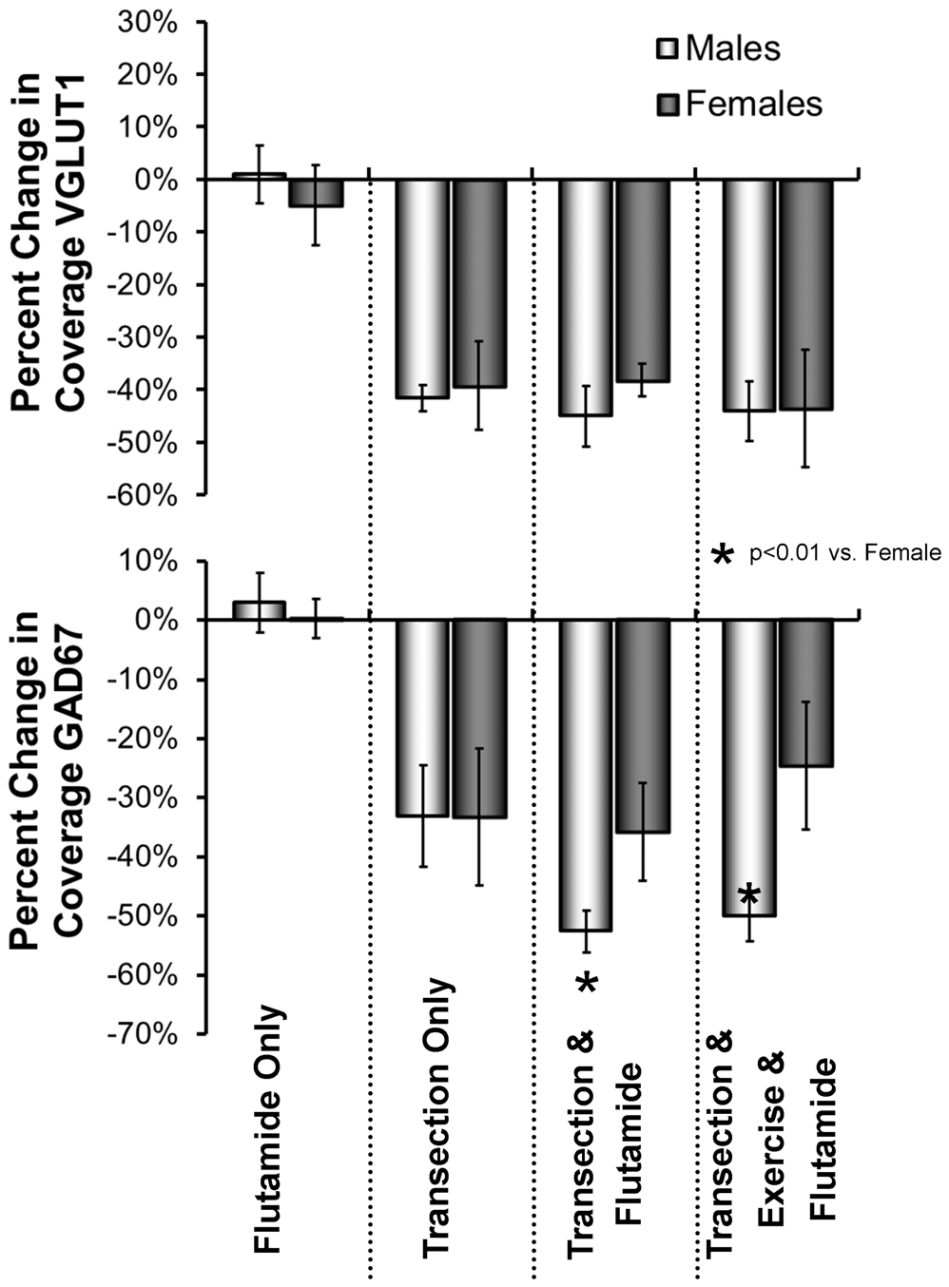
Mean (+SEM) percent change in synaptic coverage, relative to intact mice, in intact mice treated only with flutamide, mice two weeks following sciatic nerve transection that were untreated and unexercised, mice two weeks following sciatic nerve transection that were treated with flutamide only, and mice two weeks following sciatic nerve transection that were both exercised and treated with flutamide. Data are shown for males and females separately. Males were exercised with a continuous training protocol. Females were exercised with interval training. Data for VGLUT1 are in the top panel. Data for GAD67 are in the bottom panel.

We measured the effects of sex-appropriate exercise on the extent of contacts onto the somata of retrogradely labeled motoneurons two weeks after sciatic nerve transection in male and female mice that were treated with the non-steroidal anti-androgen, flutamide. As controls, we performed the same flutamide treatments in intact mice and unexercised mice whose sciatic nerves had been transected. The mean (± SEM) *percent change* in synaptic coverage by VGLUT1- IR and GAD67-IR structures in these animals, relative to that found in intact and untreated mice, is shown in Figure 4. Based on the results of ANOVA, significant differences were found for both VGLUT1 (F_9,30_ = 27.21, p<0.01) and GAD67 (F_9,30_ = 21.89, p<0.01). In intact male and female flutamide-treated mice, no significant reduction in synaptic coverage, was found relative to intact untreated mice, for either VGLUT1 or GAD67 (HSD, n.s, for both). The percent change in synaptic coverage is not significantly different from zero. Following sciatic nerve transection, a significant reduction (HSD, p<0.01) in contacts by VGLUT1+ synaptic structures or GAD67+ synaptic structures, relative to intact mice, was found in untreated mice and in flutamide-treated mice, whether or not the mice were exercised appropriately (continuous for males, interval for females). Flutamide treatment blocked the effect of exercise in both males and females.

In addition, in flutamide-treated male mice, the reduction in percent synaptic coverage by GAD67-IR structures following sciatic nerve transection was significantly (HSD, p<0.01) greater (i.e. larger negative percent change in synaptic coverage) than in flutamide-treated females, whether or not the animals were exercised. This proportional decrease in synaptic coverage in injured, flutamide treated males is also significantly greater than found in untreated and unexercised males. No significant sex differences were found in the contacts made by VGLUT1-IR structures.

## Discussion

Despite the capacity for regeneration by axons in cut peripheral nerves, poor functional outcomes after peripheral nerve injury are common [1], [2]. Slow and inefficient axon regeneration are often cited as a reason for this poor clinical outcome [31]. Changes in the circuitry of the central nervous system are considered far less often. When peripheral nerves are injured, a withdrawal of nearly half of the synaptic inputs from the somata and proximal dendrites of the axotomized motoneurons is found as well [3]. Many of these synaptic inputs are restored over time, but those arising from primary afferent neurons and expressing VGLUT1 in their terminals are not [3].

We [7], and others (e.g.[32]) have shown that moderate exercise in the form of daily treadmill training, if applied shortly after peripheral nerve injury, will promote axon regeneration. Male and female mice respond to different exercise protocols in very different ways [8]. Slow continuous walking for one hour per day is effective in enhancing axon regeneration only in males, while interval training, a series of short sprints separated by rest periods, is effective in females but not in males. Pharmacologic blocking of the androgen receptor eliminated the effectiveness of exercise in promoting axon regeneration in both sexes [18].

We studied immunoreactive contacts made by two different types of synapses onto motoneurons. Excitatory (VGLUT1+) contacts made mainly by primary afferent neurons and inhibitory (GAD67+) contacts arising from within the central nervous system were studied as models of synapses that are withdrawn from the somata of motoneurons following peripheral nerve transection. One important finding of this study is that if both male and female mice are appropriately exercised following peripheral nerve injury, the anticipated reduction in synaptic coverage on the axotomized motoneurons by both of these types of synapses is not found. The requirements for exercise to produce this effect differ dramatically between males and females. In interval trained males and continuous trained females, the coverage of the somata of axotomized motoneurons by structures immunoreactive for VGLUT1 and GAD67 is reduced to the same extent as in unexercised mice. In contrast, no significant reduction in percent synaptic coverage was found in continuous trained males and interval trained females. Thus the very same sex-dependent difference in the requirement for the effect of exercise to promote axon regeneration in the periphery also is a requirement for the effect of exercise on the withdrawal of these synaptic inputs onto axotomized motoneurons.

The cellular mechanism by which synapses are withdrawn from motoneurons following peripheral nerve transection is not well known. Initial observations [33], [34], [35], [36] were used to suggest that processes of reactive astrocytes and/or microglia, which proliferate and surround axotomized motoneurons, might play an important role, but based on the results of more recent studies [37], a shift in attention to axotomy induced changes in the motoneurons has occurred. In particular, the decline in production of cell adhesion molecules by axotomized motoneurons has been shown to precede the actual withdrawal of synapses [20], [36], [38], [39], suggesting that, in intact animals, these molecules are part of an active, retrograde signaling mechanism that promotes synapse retention. In addition, immune system-related molecules, such as the major histocompatibility complex (MHC) class I molecules [39] and members of the complement family [40] have been associated with synaptic withdrawal. In mice null for MHC class I (MHC class Ia), greater synaptic withdrawal was found after peripheral nerve transection than in wild type controls, while markedly less synaptic stripping was found after nerve injury in mice lacking complement protein C3. These findings were used to suggest that immune molecules might regulate both the shedding and retention of synapses [41]. Of considerable interest is the finding that MHC class Ia has been found in both motoneurons and microglia following peripheral nerve transection [41]. Whether our finding that no synaptic withdrawal is observed following peripheral nerve transection if mice are appropriately exercised is the result of decreased expression of complement protein C3 expression or even increased expression of MHC class I is not known at this time, but seems a fruitful avenue for future studies.

A second, and not mutually exclusive mechanism has emerged from the results of manipulation of the retrograde signaling molecule nitric oxide (NO) [42]. Peripheral axotomy results in the production of NO in somatic motoneurons, and associated reactive astrocytes, cells that do not normally produce it. Diffused NO acts directly on presynaptic terminals, activating a signaling pathway eventually leading to microtubule disruption and withdrawal. The NO also acts indirectly on the synaptic inputs. Within the axotomized motoneurons it blocks the secretion of brain derived neurotrophic factor (BDNF) at synaptic sites [43], which eventually leads to effects on cytoskeletal actin in the afferent neural terminals and mechanical destabilization of synaptic inputs. Blocking either NO production with neuronal nitric oxide synthase (nNOS) inhibitors [44] or the effects of direct downstream signaling components [45] blocks nerve crush-induced synaptic stripping. Inducing nNOS activity in motoneurons using viral constructs induces synaptic stripping in intact animals [46].

The effects of our exercise protocols on NO production following peripheral axotomy are not known, but we have shown that the effect of exercise, *both* in enhancing axon regeneration in the periphery [47] and in restoring withdrawn synaptic inputs from motoneurons [48] following peripheral nerve injury, is lost in motoneuron-specific BDNF knockout mice. Based on these observations, we suggest that one role of neuronal BDNF is to promote stabilization of synaptic inputs onto motoneurons. Consistent with this postulate, the expression of BDNF is increased transiently in motoneurons following transection of their axons and then decreases rapidly at about the time that synapses begin to be withdrawn [49]. Application of BDNF to the proximal segment of a cut eye muscle nerve restored synaptic inputs stripped from the axotomized oculomotor motoneurons [50]. It is known that treadmill training results in an increased expression of neurotrophins, especially BDNF, in spinal motoneurons [51], with a time course that is appropriate to impact synaptic withdrawal following peripheral nerve injury.

The nature of the stimulus provided by exercise that triggers this increase in BDNF expression is not clear. One means of regulation of the BDNF gene in neurons is activity [52] and the anticipated increase in activity of motoneurons found with exercise has been suggested as an explanation for the increased BDNF expression [53]. However, the finding presented above and elsewhere [18] that signaling through the androgen receptor is required for the effects of exercise on axon regeneration and synaptic stripping are consistent with a role of androgens as a stimulus to motoneuron expression of BDNF. Indeed, androgen treatments of castrated animals produced a substantial and prolonged increase in BDNF expression in facial motoneurons [54] and attenuated the amount of synaptic withdrawal after facial nerve transection [15], as well as accelerated peripheral motor axon regeneration [55]. Precisely how this combination of activity and androgens might contribute to increased expression and secretion of motoneuron BDNF is not known, nor is the relationship between BDNF signaling through the trkB receptor and the expression of cell adhesion molecules implicated in synaptic stabilization. We do not know why different exercise protocols are required in males and females. We interpret our finding that interval training in male mice and continuous treadmill training in female mice promotes *neither* enhanced axon regeneration *nor* an effect on synaptic inputs to motoneurons to mean that these forms of exercise in the different sexes do not result in sufficient combinations of increased activity and androgen availability to influence these processes. However, until we can examine the effects of a greater diversity of exercise protocols on the expression of signaling molecules such as BDNF and trkB in identified populations of motoneurons, this interpretation must remain entirely speculative.

The source of the androgens may differ in males and females. The testes are the primary source of androgens in males, and because castrating male mice blocked the effects of exercise on axon regeneration in cut nerves [8], gonadal androgens must be considered the prime candidate for the source of androgens that are required for the effects of continuous treadmill training on synaptic withdrawal in males. In females, since no significant increase in serum testosterone was noted after interval training [48], the required androgens must come from some other source. Sex steroids can be synthesized in a number of tissues, including the central nervous system [56]. In the spinal cord, enzymes in the biosynthetic pathway for androgens are found both in motoneurons [57], [58] and glial cells [59], [60], [61], [62], indicating that these cells could be a source of localized production of androgens. Of particular interest is the enzyme cytochrome C P450 aromatase, which catalyzes the conversion of testosterone and its precursors into estradiol, a potent neurosteroid. If exercise inhibited aromatase in spinal cord motoneurons and/or glial cells, an increased availability of androgens and decreased availability of estradiol might be expected in the vicinity of the axotomized motoneurons. Such localized change in expression of androgens and estrogen receptor ligands, such as estradiol, could contribute to the observed effects of exercise on synaptic inputs onto motoneurons. Such an effect is not unprecedented. Interval training in female rats resulted in a marked depression of muscle aromatase activity [63]. Pharmacologic inhibition of aromatase resulted in a marked enhancement of axon regeneration in unexercised female mice [8]. This notion could be tested by measuring or manipulating aromatase activity in motoneurons and glial cells after different forms of exercise in male and female mice.

## Conclusions

Moderate daily treadmill training following peripheral nerve transection can affect the extent of contacts made by excitatory and inhibitory synaptic inputs with the somata motoneurons. The requirements for effective exercise treatments are different for males and females. Cellular signaling through the androgen receptor is required for the effects of exercise in both sexes. Exercise could be a potent and low-technology treatment to help maintain circuitry in the central nervous system, but the requirements for its effect will require careful consideration.
